# Effect of a Diet-Induced Obesity on the Progeny Response in a Murine Model

**DOI:** 10.3390/nu15234970

**Published:** 2023-11-30

**Authors:** Maria Gallardo Paffetti, Juan G. Cárcamo, Lucía Azócar-Aedo, Angel Parra

**Affiliations:** 1 Escuela de Medicina Veterinaria, Facultad de Ciencias, Universidad Mayor, Santiago 8580000, Chile; 2 Instituto de Bioquímica y Microbiología, Facultad de Ciencias, Universidad Austral de Chile, Valdivia 5090000, Chile; gcarcamo@uach.cl; 3 Escuela de Medicina Veterinaria, Facultad de Ciencias de la Naturaleza, Universidad San Sebastián, Puerto Montt 5480000, Chile; lucia.azocara@uss.cl; 4 Facultad de Ciencias del Mar, Universidad Católica del Norte, Coquimbo 1780000, Chile; angelparra0409@gmail.com

**Keywords:** high-energy diet, maternal diet, litter size, post-weaning diet, breastfeeding

## Abstract

Diet-induced obesity could have detrimental effects on adults and their progeny. The aim of this study was to determine the effect of a high-energy diet on both F1 mice body weight and tissue/organ weight and F2 offspring growth. A simple murine model for obesity was developed using a high-energy diet and mice reared in litters of five or ten, from 30 dams receiving a cafeteria diet of either commercial chow (low energy), or a mixture of commercial chow, chocolate (50% cacao), and salty peanuts (high energy). This diet continued from mating until weaning, when the pups were allocated according to sex into eight groups based on maternal diet, litter size, and post-weaning diet. On day 74, the males were slaughtered, and the females were bred then slaughtered after lactation. As a result, the high-energy maternal diet increased the F1 offspring growth during lactation, while the high-energy post-weaning diet increased the F1 adult body weight and tissue/organ weight. The high-energy maternal diet could negatively affect the onset of the F1 but not the maintenance of breastfeeding of F1 and F2 offspring. For F2 offspring growth, the high energy overlapped the low-energy post-weaning diet, due to problems of gaining weight during lactation.

## 1. Introduction

Obesity is a multifactorial disease with high complexity, with a considerable frequency in all ages and both sexes, regardless of geographical locality, ethnicity, or socioeconomic status [1]. The prevalence of obesity has been increasing during recent years, becoming a global public-health concern [2]. It is acquiring importance during adulthood and pregnancy due to the risk factors for the mother and her offspring, such as maternal and fetal death, preeclampsia, gestational diabetes, and congenital abnormalities [3]. This has long-term consequences for the offspring, predisposing or “programming” them to develop some metabolic disease during adulthood [4,5,6], associated with chronic inflammation [7]. Maternal obesity has been postulated to induce damage in multiple fetal organs and tissues, including skeletal muscles, and the secondary consequences of exposure to an obesogenic environment in utero could severely impact postnatal life [8]. Meanwhile, it has been tested on small litter sizes and shown to promote rapid neonatal growth by overfeeding, increasing the risk of metabolic diseases during adulthood [9].

In vitro and in vivo models are important tools in studying the mechanisms of obesity, with new insights, such as discovering therapeutic targets and developing pharmacological treatment options [10]. Specifically, animal models address causality, allowing the longitudinal assessment of cardiometabolic outcomes across life courses and investigating sex differences. They can help to determine the critical windows during which offspring are particularly vulnerable to programming effects [11]. Sejrsen and Purup [12] reported that overweightness in dairy cattle causes a permanent inability of mammary glands to produce milk. It is not known whether the milk ejection reflex is affected by obesity, either in breastfeeding mothers or in animal models that will affect the normal development of the offspring. Also, different studies have shown that mice reared in small litters become obese rapidly [9].

However, although this study represents a simple neonatal model for obesity, this recent work shows that it is the energy that explains this behavior rather than the litter size. We hypothesize that a high-energy diet during lactation and after weaning influences adult body weight (BW) and tissue/organ weight, negatively affecting offspring growth in mice. This study aims to determine the effect of a high-energy diet during lactation and after weaning on F1 mice BW and tissue/organ weight, and F2 offspring growth in mice.

## 2. Materials and Methods

### 2.1. Location

The experiment was conducted in the Bioterium at the Technical University of Denmark from March to October 2015.

### 2.2. Animals and Sampling

**Figure 1:**
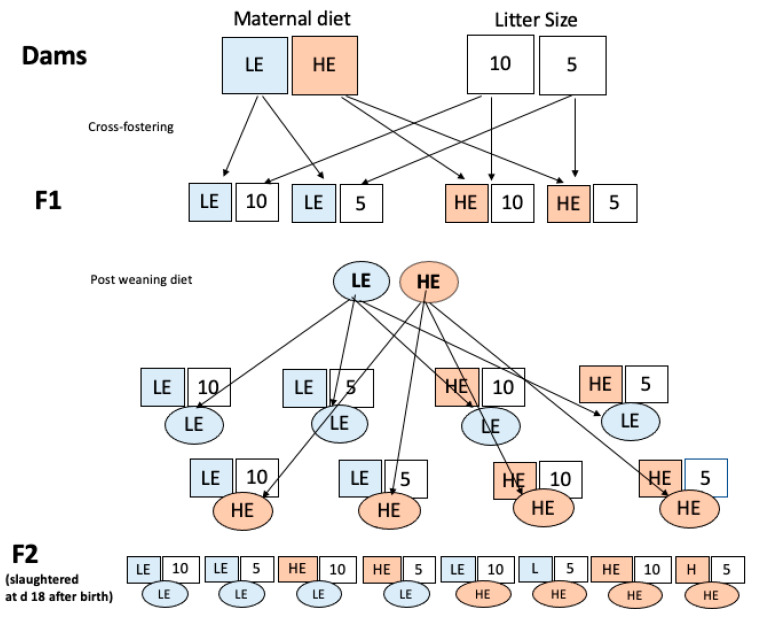
Experimental design. HE, high-energy diet; LE, low-energy diet; 10: Litter size of 10 pups, 5: Litter size of 5 pups.

Figure 1 shows the experimental design of the study. The first step included a simple model for obesity development, based on the maternal diet and litter size. Thirty NMRI female mice were subjected to 12/12 h light/darkness cycle and were randomly selected for a low-energy diet based on a standard breeding commercial chow diet (Altromin 1314 (g/100 g): 3.34 kcal/g metabolizable energy (ME), 5.1 g ether extract (EE), 50.7 g carbohydrates, and 22.5 g crude protein (CP)) and a high-energy diet (g/100 g), based on the same commercial chow, plus two energy foods frequently used in the population, such as milk chocolate 50% cacao and salty peanuts (4.7 kcal/g ME, 28 g EE, 40.6 g carbohydrates, and 18.2 g CP), in the same proportions, in a cafeteria trial, from mating at 42-days old until day 18 of lactation (at weaning).

On day 1 of lactation, the day of birth, the mice were reared in litters of ten or five balanced male and female pups, as far as possible, but cross-fostering half a litter by pups belonging to dams fed with the other diet, to eliminate or minimize the variable of direct gestational effects of maternal diet, allowing a focus on the effect of maternal diet during lactation and post-weaning diet.

Considering the female diet and litter size, we had four groups of pups: low-energy maternal diet with a litter size of ten, low-energy maternal diet with a litter size of five, high-energy maternal diet with a litter size of ten, and high-energy maternal diet with a litter size of five (F1 offspring). The first part of the study considered each F0 female included as an experimental unit.

After weaning, the pups were separated by sex, and we began to weigh pups individually 10 days later. The most obese individuals in each group were randomly selected for the second part of the study. From this subgroup, each selected 32-day-old male and female pup belonging to different groups was defined as an experimental unit based on three factors: the female diet and litter size (both shown as squares) and the post-weaning diet of the pups (shown as ovals) with eight-factor combinations (or treatments): a low-energy maternal diet with a litter size of ten and a low-energy post-weaning diet (combination 1); a low-energy maternal diet with a litter size of five and a low-energy post-weaning diet (combination 2); a high-energy maternal diet with a litter size of ten and a low-energy post-weaning diet (combination 3); a high-energy maternal diet with a litter size of five and a low-energy post-weaning diet (combination 4); a low-energy maternal diet with a litter size of ten and a high-energy post-weaning diet (combination five); a low-energy maternal diet with a litter size of five and a high-energy post-weaning diet (combination 6); a high-energy maternal diet with a litter size of ten and a high-energy post-weaning diet (combination 7); and a high-energy maternal diet with a litter size of five and a high-energy post-weaning diet (combination 8).

The F1 males were maintained on the same post-weaning diet until day 74. The F1 male BW was measured at 32, 46, 60, and 74 days old then slaughtered after the last measurement, and tissue (subcutaneous, abdominal, and perirenal fat) and organ weights (liver, left and right kidney, heart, spleen, and full gastrointestinal tract) were also collected.

The F1 female BW was measured at 32, 46, and 60 days old and bred at day 74 with males subjected to a standard diet. At birth, the F2 litter size was adjusted to ten individuals. The F2 litter pups were slaughtered on day 18 of lactation. The F1 females were maintained until slaughter (at day 18 of lactation) on the same diets introduced at weaning.

### 2.3. Statistical Design

Linear regression was used to determine the slope, analyzing the y-value (weight) and x-value (time).

For the first part of the study, a factorial model with two factors, namely maternal diet and litter size, and two levels, was used. For the second part of the study, a factorial model of three factors, namely maternal diet, litter size, and post-weaning diet, and two levels each was used. The data were analyzed through two-way and three-way ANOVA (Analysis of Variance), respectively, using the R studio statistical program (version 4.0.3). The statistical model used for the first part of the study was yijk = μ + M_i_ + L_j_ + εijk, where: yijk = observation; μ = the overall mean; M_i_ = the fixed effect of level i of maternal diet; L_j_ = the fixed effect of level j of litter size; εijk = random error. The statistical model used for the second part of the study was yijkl = μ + M_i_ + L_j_ + P_k_ + εijkl, where: yijkl = observation; μ = the overall mean; M_i_ = the fixed effect of level i of maternal diet; L_j_ = the fixed effect of level j of litter size; P_k_ = the fixed effect of level k of post-weaning diet; εijkl = random error. Results were considered significant when *p* ≤ 0.05.

## 3. Results

**Figure 2:**
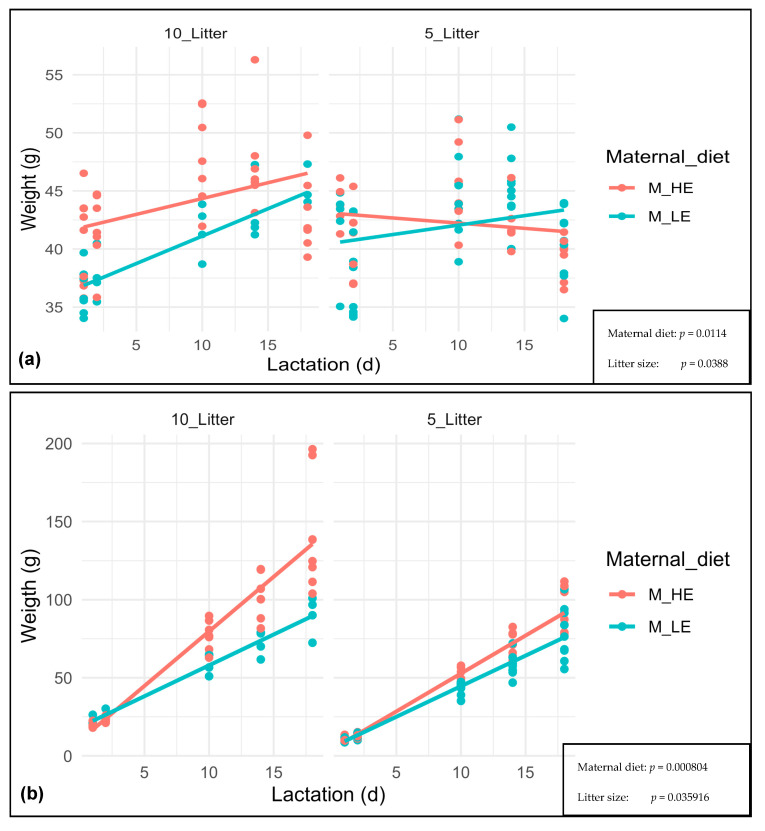
F0 maternal BW (**a**) and F1 offspring growth day 1, 2, 10, 14 and 18 of lactation (**b**). M_HE, high-energy maternal diet; M_LE, low-energy maternal diet; 10_Litter, Litter size of 10 pups; 5_Litter, Litter size of 5 pups.

The F0 maternal BW and F1 offspring growth at days 1, 2, 10, 14, and 18 of lactation is shown in Figure 2. The F0 maternal BW during lactation was more affected by the maternal diet (*p* = 0.0114) than the litter size (*p* = 0.0388). Thus, with a litter size of ten, the F0 maternal BW during lactation was increased more by a high-energy than a low-energy diet. The F0 females fed with a high-energy diet started and finished lactation with higher BW than the females fed with a low-energy diet. In the case of litter size of five, the F0 females fed with a high-energy diet showed lower BW on day 1 than on day 18 of lactation. On the other hand, the F0 females fed with a low-energy diet showed greater weight on day 1 than on day 18 of lactation.

Meanwhile, the F1 offspring growth during lactation was more affected by the maternal diet (*p* = 0.000804) than the litter size (*p* = 0.035916). The F1 offspring growth during lactation was more increased by a high-energy than a low-energy diet, especially with a litter size of ten. The F1 litters, whose F0 dams were fed with a high-energy diet during lactation, showed lower F1 offspring growth on days 1 and 2 of lactation than those whose F0 dams were fed with a low-energy diet.

**Figure 3:**
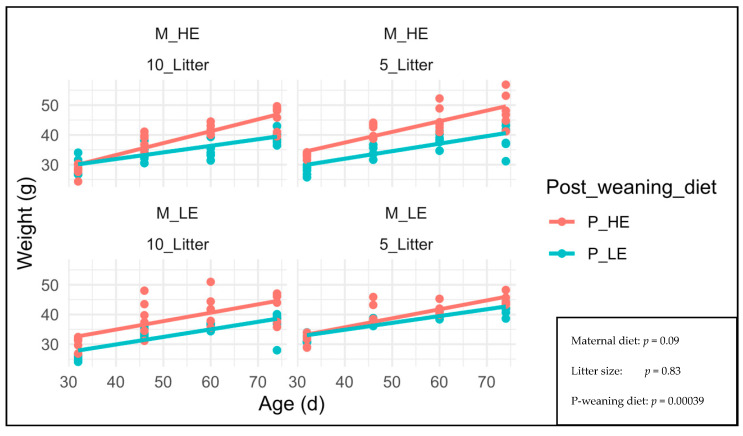
F1 male BW at 32, 46, 60 and 74 days old (age at slaughter). M_HE, high-energy maternal diet; M_LE, low-energy maternal diet; 10_Litter, Litter size of 10 pups; 5_Litter, Litter size of 5 pups; P_HE, high-energy post-weaning diet; P_LE, low-energy post-weaning diet.

Figure 3 shows the effect of maternal diet, litter size, and post-weaning diet on F1 male BW at 32, 46, 60, and 74 days old (age at slaughter).

The F1 male BW from days 32–74 was only affected by the post-weaning diet (*p* = 0.00039). The maternal diet and litter size did not affect the F1 male BW (*p* = 0.09 and 0.83, respectively). The F1 males fed with a high-energy post-weaning diet showed higher weight at 32, 46, 60, and 74 days old (age of slaughter) than those fed with a low-energy post-weaning diet.

The F1 tissue/organ weights at 74 days old (age of slaughter) showed the post-weaning diet was the only factor affecting the subcutaneous fat (*p* = 3.7 × 10^−7^), liver (*p* = 0.02), abdominal fat ((*p* = 8.46 × 10^−5^), left kidney ((*p* = 0.00275), right kidney (*p* = 8.37 × 10^−6^), spleen ((*p* = 0.0119), and full gastrointestinal weights (*p* = 0.000273). The perirenal fat weight was high in F1 males whose F0 dams were fed with a high-energy diet during lactation (*p* = 0.009) and received a high-energy post-weaning diet (*p* = 1.1 × 10^−7^), not recording any effect of the litter size (*p* = 0.71). Finally, heart weight was more affected by the post-weaning diet (*p* = 8.84 × 10^−6^) than the litter size (*p* = 0.009), without affecting the maternal diet (*p* = 0.26). Compared to those receiving a low-energy diet, the F1 males fed with a high-energy post-weaning diet showed a trend of greater tissue and organ weight, except for the full gastrointestinal, which showed a trend of lower weight in F1 males fed with a high-energy diet than those fed with a low-energy post-weaning diet (data included in Table S1).

**Figure 4:**
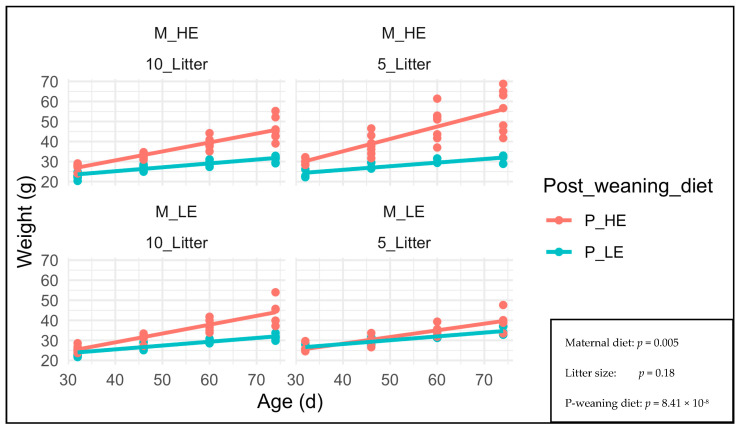
F1 female BW at 32, 46, 60 and 74 days old (age at slaughter) M_HE, high-energy maternal diet; M_LE, low-energy maternal diet; 10_Litter, Litter size of 10 pups; 5_Litter, Litter size of 5 pups; P_HE, high-energy post-weaning diet; P_LE, low-energy post-weaning diet.

Figure 4 shows F1 female BW at 32, 46, 60, and 74 days old (age at breeding). The F1 female BW was more affected by the post-weaning diet (*p* = 8.41 × 10^−8^) than the maternal diet (*p* = 0.005). The litter size had no effect on the F1 female BW (*p* = 0.18). The F1 females fed with a high-energy post-weaning diet showed greater weight at 32, 46, 60, and 74 days old than those fed with a low-energy post-weaning diet, especially if they came from F0 females fed with a high-energy diet during lactation.

The abdominal fat weight was more affected by the post-weaning diet (*p* = 2.06 × 10^−5^) than the litter size (*p* = 0.01) and maternal diet (*p* = 0.031). In the case of the left mammary gland´s weight, it was more affected by the post-weaning diet (*p* = 0.000212) than the maternal diet (*p* = 0.005769). Also, in the case of the left mammary gland’s weight, it was more affected by the post-weaning diet (*p* = 5.2 × 10^−7^) than the maternal diet (*p* = 0.00425) and litter size (*p* = 0.01). Considering the post-weaning diet on tissues/organ weight on day 18 of lactation, F1 females fed with a high-energy post-weaning diet showed a trend of greater abdominal fat and mammary gland´s weight than those F1 females fed with a low-energy post-weaning diet (data included in Table S2).

**Figure 5:**
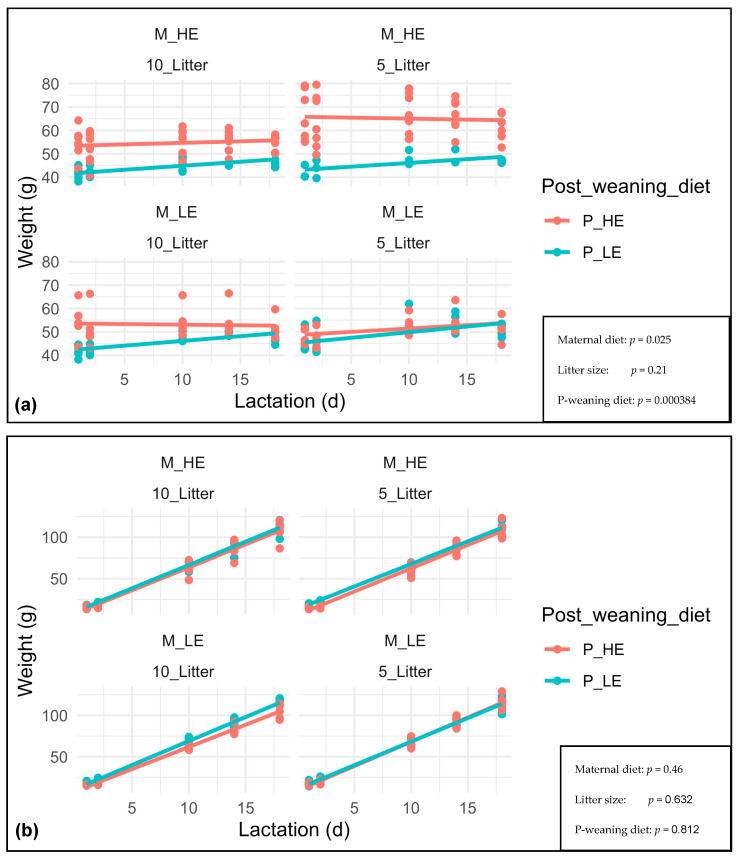
F1 females BW (**a**) and F2 offspring growth at day 1, 2, 10, 14 and 18 of lactation (**b**). M_HE, high-energy maternal diet; M_LE, low-energy maternal diet; 10_Litter, Litter size of 10 pups; 5_Litter, Litter size of 5 pups; P_HE, high-energy post-weaning diet; P_LE, low-energy post-weaning diet.

The F1 females’ BW and F2 offspring growth at days 1, 2, 10, 14, and 18 of lactation is shown in Figure 5. The F1 female BW was more affected by the post-weaning diet (*p* = 0.000384) than the maternal diet (*p* = 0.025). The litter size had no effect on the F1 female BW (*p* = 0.21). In all cases, the females fed with a high-energy post-weaning diet showed greater BW during lactation than those fed with a low-energy post-weaning diet. Regardless of the treatment, the F1 females, whose dams were fed with a high-energy diet, and those assigned to a high-energy post-weaning diet did not show any significant weight increase during lactation. Conversely, the F1 females, whose dams were fed with a low-energy diet, and those assigned to a low-energy post-weaning diet have increased their weight during lactation (data in Table S3). The F1 females fed with a high-energy diet started lactation with greater BW than that of F1 females fed with a low-energy diet (they were kept on the same diet on day 32 after weaning).

The F2 offspring growth was affected neither by the post-weaning diet (*p* = 0.812) nor by the maternal diet (*p* = 0.46) and the litter size (*p* = 0.632). Although the F2 offspring growth was increased from days 1–18 of lactation, we did not observe differences between treatments (data included in Table S3). Although the litters could have started lactation at different weights, their offspring still had similar growth after 18 days of lactation.

## 4. Discussion

Obesity constitutes one of the main problems in public health [13] regardless of animal sex. In humans, adipogenesis occurs during late pregnancy and early postnatal life [14], a sensitive process to nutrient supply in utero. Considering the low turnover of adipocytes during adulthood, the effects in utero and early postnatal life can predispose to obesity [15]. According to Segovia et al. [13], altered nutrition in the critical periods of fetal development can program alterations during organogenesis, tissue development, and metabolism, predisposing the progeny to obesity as well as metabolic and cardiovascular disorders.

Animal models for studying obesity comprise various species, from non-mammals (for example: zebrafish, *Caenorhabditis elegans*, and *Drosophila*) to mammals (rodents, large animals, and nonhuman primates) [16]. Within these, small rodents (rats and mice) are the most widely used species to assess interactions between genetic and environmental factors involved in this disease [10].

In the first part of the study, it seems that the F0 females fed with a high-energy diet and adjusted to ten pups have shown a higher BW than the females fed with a low-energy diet since days 1–18 of lactation. That could make sense, considering that they were fed with a high-energy diet from mating until day 18 of lactation, being heavier than F0 females fed with a low-energy diet. However, regardless of the slope, the individual data distribution shows a fall on day 18 of lactation. A similar trend was reported in the F0 females fed with a high-energy diet and adjusted to five pups. However, this group also showed an abrupt fall of the slope due to a sharp BW drop on day 18 of lactation. The individual data distribution F0 females fed with a low-energy diet and adjusted to litter size of five showed a similar trend of decreasing BW on day 18 of lactation.

It is also striking that the BW slope of F0 females fed with a low-energy diet and adjusted to ten pups was higher than that of those fed with a high-energy diet, increasing their BW, as explained by a correct energy supply of the low-energy diet (which strictly corresponds to a standard breeding commercial diet), formulated to meet the requirements during lactation. In the case of the high-energy diet, probably the excessive energy supply could inhibit feeding at the end of lactation as a mechanism of weight control in mice.

The F1 offspring growth during lactation was increased more by the high-energy than the low-energy maternal diet, especially in litters adjusted to ten, as shown by twice the number of pups. Considering the energy intake of the high and low-energy diets (4.7 kcal/g ME vs 3.34 kcal/g ME, respectively), it is easy to understand the higher weights found in offspring, whose F0 dams were fed with a high-energy diet, than in offspring, whose F0 dams were fed with a low-energy diet. As, in the first part of the study, the experimental unit was each F0 female, and we could not confirm the effect of the litter size on the fat growth in the offspring [9].

The F1 males fed with a high-energy post-weaning diet showed higher weight at 32, 46, 60, and 74 days old (age of slaughter) than those fed with a low-energy post-weaning diet (*p* = 0.00039), as explained by greater energy in the high-energy diet than in low-energy diet. Kulhanek et al. [17] reported that male mice exposed to a maternal high-fat and high-carbohydrate diet showed a 15% increased meal size and 46% frequency than control, without changing energy expenditure and predisposing the offspring to obesity and metabolic diseases later in life. Ito et al. [18] reported that the combination of maternal plus offspring overnutrition should predispose to oxidative stress in the offspring, accelerating metabolic syndromes.

Regarding the tissue/organ weight, the F1 males fed with a high-energy post-weaning diet showed higher weight than those fed with low-energy diets (*p* < 0.05). However, the low total gastrointestinal weight recorded for F1 males fed with a high-energy post-weaning diet (*p* = 0.000273) could be a consequence of the low digestibility of the low-energy diet [19].

In the case of the subcutaneous and abdominal adipose tissue analyzed in this study, the F1 males fed with a high-energy post-weaning diet showed a trend of higher subcutaneous and abdominal fat weight than F1 males fed with a low-energy post-weaning diet, with most reported studies agree that overweightness is associated adiposity [9]. In males, testosterone is metabolized by aromatase in fat, breast, prostate, and endothelial cells to increase the intracellular levels of estradiol necessary to activate the ER-α receptor, and therefore adipogenesis [20], increasing the subcutaneous fat deposition in hips, upper thighs, and lower abdomen, the ectopic fat deposition in muscle tissue and liver and the visceral fat deposition in the abdominal area [21,22].

Despite the significant results regarding adipose tissues, using molecular tools, such as qRT-PCR, to quantify the expression of ER-α and ER-β, where the ER-α/ER-β ratio is related to obesity and subcutaneous deposition [23], are necessary to validate these results. Also, the levels of other transcripts, such as PPAR-γ, lipoprotein lipase (LPL), adiponectin, and leptin in fetal perirenal fat have been associated with obesity in sheep [24], suggesting that maternal obesity may increase the lipogenic capacity of adipose tissue, increasing the lipid storage and hence the adiposity in the offspring [13]. In one study, rat offspring of dams fed with high-fat diets before and during pregnancy (but not lactation) developed alterations in the plasma and adipose tissue, such as elevated circulating triglycerides despite de novo lipogenesis [10]. Additional studies should determine which adipose tissue was the most affected by the high-energy diets in mother and offspring, considering its deepness in the body and the melting points of the constituent fatty acids [25].

It has been reported that a high-fat diet, given during pregnancy and lactation in mice, could increase heart and kidney weight and reduce cardiac insulin responsiveness, expanding the risk of cardiorenal diseases in male mice [26]. However, our results showed the heart weight was more affected by the post-weaning diet (*p* = 8.84 × 10^−6^) than the litter size (*p* = 0.009), not showing any significant effect of the maternal diet (*p* = 0.26). Finally, although in our study the kidney weight was only affected by the post-weaning diet, and the perirenal adipose tissue weight was high in F1 males fed with a high-energy post-weaning diet, especially when they came from F0 dams fed with a high-energy diet during lactation, which also was affected by the maternal diet [26].

Following the same trend reported for F1 males’ BW, the F1 females fed with a high-energy post-weaning diet showed greater weight at 32, 46, 60, and 74 days old than those fed with a low-energy post-weaning diet (*p* = 8.41 × 10^−8^), especially if they came from F0 females fed with a high-energy diet during lactation, which affected the maternal diet (*p* = 0.005). This is attributed to the increased energy intake from the maternal and post-weaning diets.

Considering the greatest *p*-value of the post-weaning diet on tissue/organ weights on day 18 of lactation (*p* = 2.06 × 10^−5^), the F1 females fed with a high-energy post-weaning diet showed a trend of greater abdominal fat and mammary gland weight than those F1 females fed with a low-energy post-weaning diet, as explained by the EE and ME proportions included in both diets. Compared to the low-energy diet, it was noticed that the high-energy diet provided more fat (28 vs. 5.1 g EE, respectively) and energy supply (4.7 vs. 3.34 kcal/g ME, respectively), increasing the fat content and the adipose tissue and mammary gland weight. Subbaramaiah et al. [27] reported that obesity led to inflammation in the mammary gland, in addition to visceral fat, increasing the expression of mRNA for aromatase, and therefore circulating estrogens in mice, delaying milk production. These parameters are not included in the present study, but, it must be tested in the future.

Another factor to consider is the effect of a high-energy diet on breastfeeding. In animals, contrary to humans, breastfeeding is a natural behavior performed by instinct [28]. It has been reported that the onset and maintenance of breastfeeding are affected negatively by obesity [29,30,31]. In the present study, F1 and F2 offspring-maintained breastfeeding, as measured by the litter’s ability to gain weight steadily until day 18 of lactation.

Although the maintenance of breastfeeding reached high F1 offspring growths when the F0 dams were fed with a high-energy rather than low-energy diet and adjusted to a litter size of ten pups, it seems to be a physiological process, independent of any factor considered in this study. In fact, we could observe that although all offspring had steadily grown during the first 10–12 days of lactation, some of the F0 dams lost weight and died, perhaps due to the inability to overcome their negative energy balance. This is consistent with the no significant results reported by the F2 offspring growth. Although the F1 dams fed with a high-energy post-weaning diet showed no significant weight increase during lactation (showing similar slopes), the F2 offspring grew steadily along lactation.

It was noticed that the F1 dams fed with a low-energy post-weaning diet showed a trend of increased BW along lactation (*p* = 0.000384). This result was confirmed by the significant F1 dams’ weight slopes *p*-values, especially when the maternal and post-weaning diets were low-energy (0.66 ± 0.19 and 0.76 ± 0.13, respectively). Thus, the non-significant F2 offspring growth slopes would reflect the low-energy diet, which strictly corresponds to a standard breeding commercial diet, being overlapped by the high-energy diet due to difficulties of gaining weight during lactation, as shown by the F1 dams fed with a high-energy post-weaning diet.

Despite the F0 females fed with a high-energy diet lactating more heavily than F0 females fed with a low-energy diet (they were kept on the same diet from mating), their F1 offspring growth at days 1 and 2 of lactation was lower than the F1 offspring growth, whose F0 dams were fed with a low-energy diet. Thus, considering the offspring growth during lactation, the high-energy maternal diet could negatively affect the onset of breastfeeding of the F1 offspring but not the maintenance of breastfeeding of the F1 and F2 offspring. It has been reported that maternal obesity in humans is related to increased intrauterine growth restriction [32,33]. In the present study, it should be supported by the lower F1 offspring growth in the first 2 days of lactation. As reported by Flint et al. [34], working on diet-induced obesity in mice, a delay in lactogenesis was observed, which relates to prolactin resistance because of elevated leptin levels [35].

A relationship has been reported between obesity induced by a high-fat diet during pregnancy and the metabolic syndrome predisposition, regardless of other factors, such as environment or the postnatal diet [36,37]. In humans, a high body mass index before and during early pregnancy predicts high birth-weight babies and a higher risk of developing metabolic syndrome [38,39,40,41]. Conversely, in the present study, a high body mass before and in early pregnancy could indicate low offspring growth at birth until day 2 of lactation. There is evidence of tissue-specific impairments in the offspring from obese dams [42,43]. Thus, deficiencies in insulin-sensitive tissues, such as adipose tissue, pancreas, liver, and skeletal muscle, may trigger insulin resistance and type 2 diabetes in the offspring [13].

Thus, knowing the pulsatile prolactin secretion stimulated by suckling is essential to establish lactation in most species. Rasmussen and Kjolhede [44] suggested a negative relationship between maternal obesity and suckling-related prolactin secretion during the first week in humans, delaying the first 24 h of spontaneous prolactin release (without any relationship with the progesterone concentration) and the onset of the milk breastfeeding, without considering the adverse effects on the fetal viability.

In the present study, a relationship between obesity and a possible lack of maternal behavior was observed in the F1 dams, being expressed as killing but not eating 1, 2, or all the pups, killing and only eating 1, 2, or all pups during the first 10 days of lactation (excluding a natural dam selection for a lack or low ability to survive of the pups). The lack of nesting behavior and protective maternal instinct for the pups when they were manipulated was impossible to measure statistically because that was unreported. Therefore, it is not considered in the hypothesis. In this respect, Bellisario et al. [45] reported that a maternal high-fat diet during pregnancy should have detrimental effects on the dam’s behavior, leading to cannibalistic behavior during this period. They suggested that a maternal high-fat diet acts as a stressful challenge during pregnancy, impairing the neuroendocrine system and the neural activity of brain regions involved in processing relevant olfactory stimuli, with negative consequences leading to aberrant maternal behavior, such as aggression [46].

According to a report by Yan et al. [47], obesity could increase adiposity in the offspring as a product of fetal inflammation. Thus, the intense offspring growth reached by F1 litters during lactation, whose F0 dams were fed with a high-energy diet and adjusted to a litter size of ten, could be explained by increased adiposity as a product of fetal inflammation during pregnancy.

Exposure to the influences of maternal obesity during the mice development could lead to adult progeny, with cardiovascular adiposity and metabolic dysfunction, hyperphagia, physical inactivity, and altered adipocyte metabolism [48]. Thus, maternal obesity can induce an abnormal body-fat deposition in the offspring, predisposing them to obesity during adulthood, as in the F1 males and females.

Regarding organs, exposing the fetal liver to triglycerides, lipids, and adipokines increases the expression of lipogenic genes, such as sterol regulatory element binding protein-lc (SREBP-1c), resulting in hepatic lipid accumulation [49] and steatosis in the offspring [48,50], especially if they were fed with a high-fat diet during the pregnancy and lactation [51,52,53]. Thus, despite the fetal programming effects, they can be amplified using a poor-quality diet during postnatal life [54]. Therefore, further studies should be designed to accommodate the lifetime of the pups from gestation until adulthood. To implement an effective risk-reduction strategy for overweight and obesity, the individual and population levels must know the factors influencing their development [55].

## 5. Conclusions

The F1 offspring growth during lactation was increased more by the high-energy than the low-energy diet maternal diet. The high-energy post-weaning diet increased the F1 male BW from days 32 to 74 (age of slaughter), including tissue/organ weights, except the heart, which showed the effects of litter size, and the perirenal fat, which showed the effects of the maternal diet. In F1 females, the high-energy post-weaning diet was the most important factor influencing the F1 female BW from days 32 to 74 (age of breeding), including the abdominal fat and both mammary glands.

The high-energy maternal diet could negatively affect the onset of breastfeeding of the F1 offspring but not the maintenance of breastfeeding of F1 and F2 offspring, which seems to be a physiological process independent of any variation factor considered in this study.

The F1 dams BW, along with the lactation of F2 offspring, was influenced more by the post-weaning than the maternal diet. Although none of the three factors considered in this study influenced the F2 offspring growth, the low-energy diet, which strictly corresponded to the standard breeding commercial diet, was overlapped by the high-energy diet, probably due to difficulties of gaining weight, as shown by the F1 dams fed high-energy post-weaning diet along lactation.

These results could open a new research line in human health due to the high prevalence and incidence of overweight and obesity worldwide. It is necessary to increase awareness regarding maternal nutrition in pregnancy, during breastfeeding, and after weaning through consultation and personalized advice for each patient/user by health professionals. This would prevent future diseases in the progeny, which will increase the life quality of people and reduce epidemiological indicators, such as Disability Adjusted Life Years (DALYs), morbidity, and mortality due to noncommunicable diseases, such as obesity.

## Data Availability

The data can be found in the supplementary materials file S1.

## Supplementary Materials

The following supporting information can be downloaded at: https://www.mdpi.com/article/10.3390/nu15234970/s1, Table S1. Subcutaneous, abdominal and perirenal fat weight, plus liver, left and right kidneys, heart, spleen and full gastrointestinal weight (LSM ± SEM) in F1 males at 74 days old (age at slaughter); Table S2. Abdominal fat plus left and right mammary glands weights (LSM ± SEM) in F1 female at day 18 of lactation of F2 (age of slaughter); Table S3. F1 female body weight and F2 offspring growth slopes *p*-values (LSM ± SEM) during lactation.
