# Supplementary material for: Effect of a Diet-Induced Obesity on the Progeny Response in a Murine Model
Source: Nutrients. 2023 Nov 30;15(23):4970. doi: 10.3390/nu15234970 (PMC10708177; doi:10.3390/nu15234970)
Supplement: Supplementary file 1 [file nutrients-15-04970-s001.zip › nutrients-2632981-supplementary.pdf]

Supplementary files

Table S1. Subcutaneous, abdominal and perirenal fat weight, plus liver, left and right kidneys, heart, spleen and full gastrointestinal weight (LSM ± SEM) in F1 males at 74 days old (age at slaughter)

**Post weaning diet**	**P_HE**	**P_HE**	**P_HE**	**P_HE**	**P_LE**	**P_LE**	**P_LE**	**P_LE**
**Maternal diet**	**M_HE**	**M_HE**	**M_LE**	**M_LE**	**M_HE**	**M_HE**	**M_LE**	**M_LE**
**Litter size**	**10_litter**	**5_litter**	**10_litter**	**5_litter**	**10_litter**	**5_litter**	**10_litter**	**5_litter**
	**(n=6)**	**(n=6)**	**(n=6)**	**(n=5)**	**(n=6)**	**(n=6)**	**(n=6)**	**(n=6)**
Subcutaneous fat	1.08 ± 0.14	1.10 ± 0.14	1.04 ± 0.14	0.79 ± 0.16	0.48 ± 0.14	0.42 ± 0.14	0.48 ± 0.14	0.29 ± 0.14
Abdominal fat	1.62 ± 0.29	1.94 ± 0.29	1.50 ± 0.29	1.15 ± 0.31	0.76 ± 0.29	0.66 ± 0.29	0.73 ± 0.29	0.60 ± 0.29
Perirenal fat	0.41 ± 0.07	0.59 ± 0.07	0.35 ± 0.07	0.27 ± 0.07	0.13 ± 0.07	0.14 ± 0.07	0.07 ± 0.07	0.05 ± 0.07
Liver	2.15 ± 0.11	2.17 ± 0.11	2.29 ± 0.11	2.43 ± 0.12	1.89 ± 0.11	2.12 ± 0.11	1.95 ± 0.11	2.34 ± 0.11
Left kidney	0.34 ± 0.02	0.34 ± 0.02	0.33 ± 0.02	0.36 ± 0.02	0.30 ± 0.02	0.32 ± 0.02	0.30 ± 0.02	0.30 ± 0.02
Right kidney	0.36 ± 0.02	0.38 ± 0.02	0.39 ± 0.02	0.36 ± 0.02	0.30 ± 0.02	0.35 ± 0.02	0.29 ± 0.02	0.31 ± 0.02
Heart	0.33 ± 0.02	0.34 ± 0.02	0.28 ± 0.02	0.33 ± 0.02	0.25 ± 0.02	0.27 ± 0.02	0.24 ± 0.02	0.30 ± 0.02
Spleen	0.24 ± 0.03	0.24 ± 0.03	0.22 ± 0.03	0.29 ± 0.03	0.17 ± 0.03	0.22 ± 0.03	0.18 ± 0.03	0.21 ± 0.03
Full gastrointestinal	5.01 ± 0.29	5.10 ± 0.29	4.82 ± 0.29	5.43 ± 0.32	5.91 ± 0.29	6.13 ± 0.29	5.41 ± 0.29	6.08 ± 0.29

P_HE, High energy post weaning diet; P_LE, Low energy post weaning diet; M_HE, High energy maternal diet; M_LE, Low energy maternal diet; 10_litter, litter adjusted to 10 pups; 5_litter, litter adjusted to 5 pups.

Table S2. Abdominal fat plus left and right mammary glands weights (LSM ± SEM) in F1 female at day 18 of lactation of F2 (age of slaughter)

**Post weaning diet**	**P_HE**	**P_HE**	**P_HE**	**P_HE**	**P_LE**	**P_LE**	**P_LE**	**P_LE**
**Maternal diet**	**M_HE**	**M_HE**	**M_LE**	**M_LE**	**M_HE**	**M_HE**	**M_LE**	**M_LE**
**Litter size**	**10_litter**	**5_litter**	**10_litter**	**5_litter**	**10_litter**	**5_litter**	**10_litter**	**5_litter**
	**(n=7)**	**(n=7)**	**(n=5)**	**(n=5)**	**(n=6)**	**(n=3)**	**(n=6)**	**(n=6)**
Abdominal fat	1.10 ± 0.31	2.85 ± 0.31	1.27 ± 0.36	1.04 ± 0.36	0.12 ± 0.33	0.20 ± 0.47	0.13 ± 0.33	0.37 ± 0.33
Left glands	0.74 ± 0.06	0.92 ± 0.06	0.73 ± 0.07	0.62 ± 0.07	0.60 ± 0.07	0.51 ± 0.10	0.44 ± 0.02	0.56 ± 0.07
Right glands	0.73 ± 0.05	0.96 ± 0.05	0.71 ± 0.06	0.61 ± 0.06	0.45 ± 0.05	0.52 ± 0.08	0.43 ± 0.05	0.53 ± 0.05

P_HE, High energy post weaning diet; P_LE, Low energy post weaning diet; M_HE, High energy maternal diet; M_LE, Low energy maternal diet; 10_litter, litter adjusted to 10 pups; 5_litter, litter adjusted to 5 pups.

Table S3. F1 female body weight and F2 offspring growth slopes p-values (LSM ± SEM) during lactation

**Post weaning diet**	**P_HE**	**P_HE**	**P_HE**	**P_HE**	**P_LE**	**P_LE**	**P_LE**	**P_LE**
**Maternal diet**	**M_HE**	**M_HE**	**M_LE**	**M_LE**	**M_HE**	**M_HE**	**M_LE**	**M_LE**
**Litter size**	**10_litter**	**5_litter**	**10_litter**	**5_litter**	**10_litter**	**5_litter**	**10_litter**	5_litter
F1 female BW	0.24 ±0.33	0.23 ±0.46	0.12 ±0.26	0.56 ±0.29	0.49 ±0.20	0.54 ±0.05	0.66 ±0.19	0.76 ±0.13
F2 offspring growth	5.69 ±0.59	5.81±0.40	5.58 ±0.48	6.02 ±0.48	5.73 ±0.50	5.47 ±0.49	5.89 ±0.28	5.76 ±0.48

P_HE, High energy post weaning diet; P_LE, Low energy post weaning diet; M_HE, High energy maternal diet; M_LE, Low energy maternal diet; 10_litter, litter adjusted to 10 pups; 5_litter, litter adjusted to 5 pups.
